# Supplementary material for: Assessing Impacts of Additives on Particulate Matter and Volatile Organic Compounds Produced from the Grilling of Meat
Source: Foods. 2022 Mar 14;11(6):833. doi: 10.3390/foods11060833 (PMC8953238; doi:10.3390/foods11060833)
Supplement: Supplementary file 1 [file foods-11-00833-s001.zip › foods-1480614-supplementary.pdf]

Supplementary data

1. The experimental data moisture content, lipid content and protein content were shown in Table S1. The addition of additives significantly affected the moisture content of meat.

Table S1. Comparison of chemical composition of pork belly marinaded with various additives

Chemical composition	Raw pork	Additive types			
		Salt	White pepper	Garlic powder	Mixed spices
Moisture content	71.25±1.31[a]	64.57±1.23[b]	61.51±0.34[c]	68.47±1.82[a]	65.74±1.01[b]
Fat content	6.84±1.93[a]	5.87±0.85[b]	6.56±1.01[a]	6.83±1.29[a]	6.64±1.83[a]
Protein content	20.21±0.99[a]	19.55±1.53[a]	19.83±1.17[a]	19.01±1.03[a]	19.36±0.87[a]

2. Figure S1 shows that the average total particulate matter (TPM) mass concentration emitted from the meat-grilling process, and the TPM mass concentration was determined to be 40.47±5.16 mg/m^3 (control group), 21.1 ±3.52 mg/m^3 (salt), 14.13±4.09 mg/m^3 (white pepper), 27.17±2.97 mg/m^3 (garlic powder), and 17.8±0.95 mg/m^3 (MS), respectively. The meat without any additives marinade generated higher (p < 0.05).

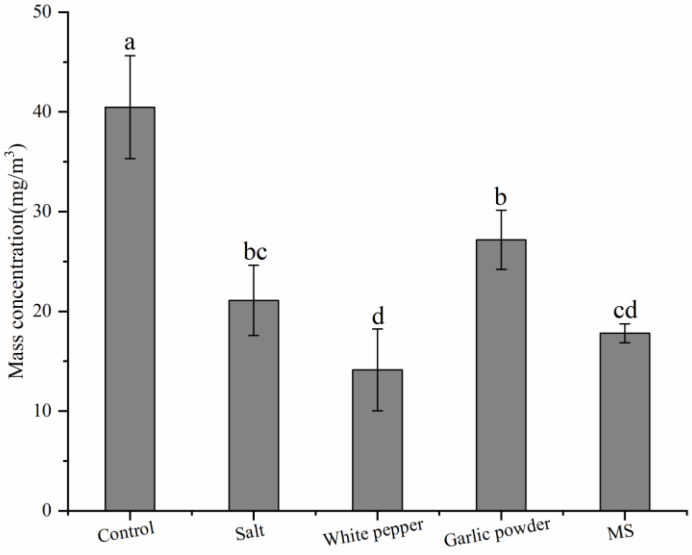

Figure S1. Total particulate matter mass concentration produced from the grilling of meat marinated with different additives. Different lowercase letters above each bar means significant differences. Error bar denotes standard deviation.
